# The Influence of Gender and Professional Background on the Accuracy of Visual Blood-Loss Estimation in Obstetrics—Prospective Observational Simulation Study

**DOI:** 10.3390/jcm15135142

**Published:** 2026-07-01

**Authors:** Maximilian Niederer, Mathias Bader, Chiara Archam, Sascha Hammer, Sebastian Labenbacher, Helmar Bornemann-Cimenti, Lioba Heuschneider, Philipp Zoidl

**Affiliations:** Department of Anesthesiology and Intensive Care Medicine, Medical University of Graz, Auenbruggerplatz 5, 8036 Graz, Austria

**Keywords:** blood loss, surgical, postpartum hemorrhage, sex characteristics, hemorrhage/diagnosis, clinical decision making

## Abstract

**Background/Objectives**: Accurate visual estimation of blood loss during childbirth is critical for early recognition of obstetric hemorrhage, a leading cause of maternal morbidity and mortality. Despite its widespread use, visual estimation is prone to substantial bias. While professional experience has been shown to influence estimation accuracy, the potential contribution of gender differences remains insufficiently explored. **Methods**: We carried out a prospective observational simulation-based study at a tertiary university medical center in Graz, Austria, to determine whether gender and professional background are associated with differences in the accuracy of visual blood-loss estimation in obstetric bleeding. Fifty physicians (28 females/22 males) were recruited from anesthesiology and obstetrics. All participants visually estimated blood loss in four simulated obstetric hemorrhage scenarios. The simulated blood volumes and hemoglobin concentrations were verified by volume measurement and point-of-care testing. Each scenario was viewed under standardized conditions without access to physiological or contextual clinical information. The primary outcome was absolute estimation error (mL) according to the gender or professional background of the participants. Secondary outcomes included scenario-specific accuracy and the association between self-rated confidence and estimation accuracy. **Results**: Women outperformed men in low and moderate-volume scenarios. Across all scenarios combined, women demonstrated lower median absolute estimation errors, although the overall difference did not reach statistical significance. Professional background showed a stronger effect: gynecologists were significantly more accurate than anesthetists across most scenarios. **Conclusions**: Visual blood-loss estimation accuracy in obstetric simulations is influenced by both gender and professional background. Gender-related differences appear volume-dependent, whereas professional experience exerts consistent influence.

## 1. Introduction

Accurate estimation of blood loss during obstetric surgery is essential for the timely recognition and management of obstetric hemorrhage, one of the leading global causes of maternal morbidity and mortality. Clinicians worldwide rely on estimated blood loss (EBL) to guide critical decisions, including thresholds for transfusion, fluid management strategies, and the activation of postpartum hemorrhage (PPH) protocols. Visual estimation remains the most commonly used method worldwide, even though extensive evidence shows that it is prone to inaccuracy and bias. While being subject to wide interindividual variation, research shows patterns of underestimation at low volumes and overestimation at high volumes [1,2,3]. Obstetric environments further complicate estimation accuracy because blood often mixes with amniotic fluid, irrigation solutions, and other serosanguinous fluids, altering visual appearance and reducing perceptual reliability [4]. An emerging area of interest concerns how gender-related differences in visual and perceptual processing may influence EBL accuracy. Although the clinical literature addressing gender differences in diagnostic or perceptual accuracy remains limited, a substantial body of foundational work in visual cognition demonstrates that women and men may process visual information in systematically different ways. Differences have been documented in contrast sensitivity, fine-grained color discrimination, luminance processing, and susceptibility to visual illusions [5]. The experimental findings indicate that gender-related variation extends to visuospatial memory integration and the reconstruction of complex visual scenes, particularly when the interpretation of subtle cues is required [6]. These perceptual distinctions suggest that gender could contribute to variation in clinically relevant tasks such as visual blood-loss estimation [7]. Despite these theoretical hints, gender-specific differences in EBL accuracy have received little attention in previous research. Most EBL studies either collapse clinician characteristics altogether or focus exclusively on the general inaccuracy of visual estimation, overlooking gender as a potential determinant. To our knowledge, no earlier investigation has evaluated whether women and men differ in their ability to estimate blood loss across distinct obstetric bleeding conditions or whether any observed differences vary with the magnitude of blood loss. Professional background represents another important yet insufficiently explored factor that potentially influences EBL accuracy. Obstetricians, due to frequent exposure to intraoperative bleeding, may develop refined perceptual calibration through repeated practice. Existing research demonstrates that obstetric surgeons tend to outperform clinicians who rely less on operative visual cues, such as anesthetists, who typically integrate physiological parameters into their assessment [2]. Simulation studies support these observations, showing that estimation accuracy varies by specialty; however, no studies have explored whether gender interacts with professional background or whether both factors contribute independently to performance [8,9]. Additionally, to date, no study has simultaneously evaluated gender as the primary determinant of EBL performance while accounting for professional background and the differing perceptual demands imposed by low versus high-volume hemorrhage. This represents a knowledge gap, as different bleeding volumes may emphasize different aspects of visual processing. Given the central role of accurate blood-loss estimation in obstetric safety, and the theoretical and empirical basis for both gender and profession-related perceptual differences, there is a need to clarify how these factors jointly influence EBL accuracy. A more nuanced understanding of clinician characteristics that affect perceptual judgement could strengthen interdisciplinary communication, support earlier recognition of hemorrhages, and help reduce delays in escalation of care. This present study addresses these gaps by systematically examining gender differences in visual blood-loss estimation across four obstetric hemorrhage simulation scenarios. Gender was conceptualized as the primary variable of interest, with professional background assessed as a secondary factor. By evaluating clinician performance across both low and high-volume bleeding scenarios, this study aimed to identify when gender-related differences are most pronounced and how professional experience contributes to the accuracy of visual estimation.

## 2. Materials and Methods

This prospective observational simulation study was conducted at the Medical University of Graz in November 2025. Simulations were performed on 5 November 2025. This study followed institutional guidelines for human research, complied with the Declaration of Helsinki, and received ethical approval from the institutional review board (IRB number 1269/2025) of the Medical University of Graz on 3 November 2025. All participants provided written informed consent prior to enrollment.

Fifty clinicians were voluntarily recruited from the departments of Anesthesiology and Obstetrics and Gynecology. Eligibility criteria required at least three months of clinical experience in operative obstetrics or obstetric anesthesia. Individuals with known color-vision deficiency were excluded. Sample size was based on an a priori power analysis designed to detect a difference of 40 mL in estimated blood loss between female and male participants at a two-sided significance level of 5% and a power of 80%. These calculations resulted in 50 required participants, with equal group sizes. This sample size is consistent with previous simulation-based studies on visual blood-loss estimation, which have used comparable numbers of participants and demonstrated sufficient power to detect clinically meaningful differences.

Four standardized obstetric hemorrhage scenarios were created using mixtures of packed red blood cells and isotonic saline solution to simulate realistic intraoperative bleeding. True hemoglobin concentrations were verified using point-of-care blood gas analysis to ensure credible color and dilution profiles.

▪ Scenario 1 (Caesarean birth): True simulated blood loss of 438 mL, Hb 10.9 g/dL; low-volume, dark, concentrated appearance representing early intraoperative bleeding.
▪ Scenario 2 (Ectopic pregnancy): True simulated blood loss of 811 mL, Hb 9.4 g/dL; moderate-to-high volume, with brighter, diluted appearance mimicking mixed intra-abdominal bleeding.
▪ Scenario 3 (Placenta previa): True simulated blood loss of 622 mL, Hb 8.6 g/dL; mid-range volume with moderate dilution.
▪ Scenario 4 (Postpartum hemorrhage): True simulated blood loss of 1014 mL, Hb 6.5 g/dL; high-volume, visually saturated appearance representing severe PPH.

Simulated blood was presented in obstetric suction canisters and medical swabs commonly used during obstetric operations. The scenarios were prepared and rechecked according to the scenario photos, which can be viewed in the Supplementary Material. Each participant viewed all four scenarios individually under standardized indoor lighting. From a viewing distance of 1.50 m, participants had 15 s to visually estimate blood loss. No hemodynamic parameters or additional contextual information were provided, ensuring reliance solely on visual assessment. The order of the scenarios was random, and the scenarios were shuffled between participants.

### Outcome Measures

The primary outcome was the absolute estimation error in mL (absolute difference between visually estimated and true blood volume in the simulated scenario).

Secondary outcomes included:

▪ Scenario-specific estimation accuracy.
▪ Direction of error (underestimation vs. overestimation).
▪ Variability in estimation patterns (interquartile range).
▪ Exploratory subgroup analyses: gender, professional background, menstrual status, age, years of experience.

Gender was the primary independent variable. Professional background (anesthesiology vs. gynecology) was evaluated as a secondary predictor.

Analyses were performed using IBM SPSS Statistics Version 29. Normality of continuous variables was assessed using Kolmogorov–Smirnov and Shapiro–Wilk tests. As absolute estimation errors were non-normally distributed, non-parametric tests were applied. Between-group comparisons used Mann–Whitney U tests for continuous variables and chi-square tests for categorical variables. Descriptive statistics are presented as medians and IQR unless indicated otherwise. Statistical significance was defined as *p* < 0.05 (two-sided). Subgroup analyses were exploratory, with no correction for multiple testing.

Generative artificial intelligence (ChatGPT-5.1 Instant) has been used in this paper to generate a draft of the text and graphics.

## 3. Results

A total of 50 clinicians participated in the study: 28 women (55%) and 22 men (45%). Professional backgrounds were evenly distributed, with 25 anesthetists and 25 gynecologists participating. The median clinical experience was 6.0 years (IQR 7.5), indicating a mix of early-career and more experienced clinicians. Female participants were additionally asked to report menstrual status: 23 women (82%) reported that they were currently undergoing a menstrual cycle.

The mean self-rated confidence in visual blood-loss estimation across all four scenarios was 2.6 ± 1.0 on a 5-point Likert scale, suggesting moderate perceived certainty. The baseline characteristics are summarized in Table 1.

### 3.1. Gender Differences in Estimation Accuracy Across Scenarios

Across all four scenarios combined, women demonstrated lower median absolute estimation errors than men. Although this overall gender difference did not reach conventional statistical significance (Mann–Whitney U = 216.0, *p* = 0.072), women showed a narrower interquartile range and fewer high-error outliers, indicating more consistent and tightly clustered performance. In contrast, male participants displayed a greater dispersion of error values and several extreme overestimation episodes.

**Figure 1:**
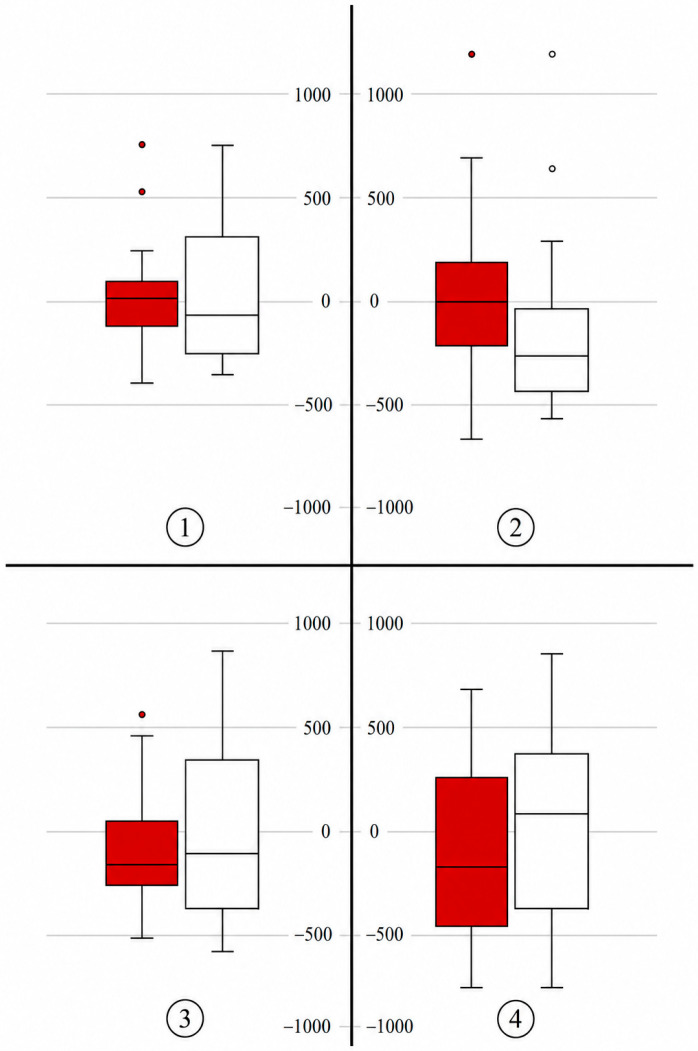
Estimated blood loss error provided by female (red) and male (white) participants across four simulated clinical scenarios. ① Scenario 1: Caesarean birth; ② Scenario 2: ectopic pregnancy; ③ Scenario 3: placenta previa; ④ Scenario 4: postpart. hemorrhage.

Figure 1 provides a detailed overview of gender-specific estimation accuracy for each of the four simulation scenarios separately.

▪ Scenario 1 (low volume): Women estimated blood loss significantly more accurately than men, with lower median errors (88 mL vs. 225 mL; median absolute error, female vs. male) and a visibly narrower spread. This scenario, representing early intraoperative hemorrhage with a relatively small volume, showed the clearest gender separation.
▪ Scenario 2 (intermediate–high volume): Gender differences in this scenario were smaller and not as pronounced as in Scenario 1 (211 mL vs. 311 mL; median absolute error, female vs. male). Both women and men showed increased variability compared with the low-volume condition.
▪ Scenario 3 (moderate volume): Women demonstrated superior accuracy, with lower median errors and fewer outliers (150 mL vs. 300 mL; median absolute error, female vs. male). This scenario combined a moderate volume with mixed visual characteristics.
▪ Scenario 4 (high volume): There was no significant gender variability in this scenario (375 mL vs. 350 mL; median absolute error, female vs. male). Women and men showed converging median error values, indicating that gender-related performance differences were less apparent at high blood-loss volumes.

In summary, these findings suggest that gender differences in visual blood-loss estimation are most pronounced under low and moderate-volume conditions (Scenarios 1 and 3), while performance becomes more similar between women and men as bleeding volume increases (Scenario 4).

### 3.2. Effect of Professional Background on Overall Accuracy

Professional background exerted a stronger and statistically robust influence on estimation accuracy than gender. When all four scenarios were pooled, gynecologists significantly outperformed anesthetists. Gynecologists had lower median absolute estimation errors and considerably less variability in their performance compared with anesthetists (Mann–Whitney U = 126.0, *p* < 0.001).

Across all scenarios combined, gynecologists demonstrated a more compact distribution of absolute estimation errors, characterized by lower median values, a narrower interquartile range, and only a small number of moderate outliers. In contrast, anesthetists showed a markedly wider dispersion of errors, including several extreme values indicative of substantial overestimation. These large deviations were primarily driven by performance in the higher-volume scenarios, where absolute estimation errors were frequently pronounced.

Scenario-specific analyses supported this overall pattern. Gynecologists were significantly more accurate than anesthetists in Scenarios 1, 3, and 4, with the largest divergence evident in Scenario 3, where female gynecologists exhibited the lowest errors of all subgroups and male anesthetists the highest. These scenario-specific comparisons are summarized in Table 2.

### 3.3. Confidence-Accuracy Relationship by Gender

At the cohort level, self-rated confidence in visual blood-loss estimation showed only a weak and statistically non-significant association with mean absolute estimation error (Spearman ρ = 0.215, *p* = 0.129). However, when the analysis was stratified by gender, a strikingly different pattern emerged.

**Figure 2:**
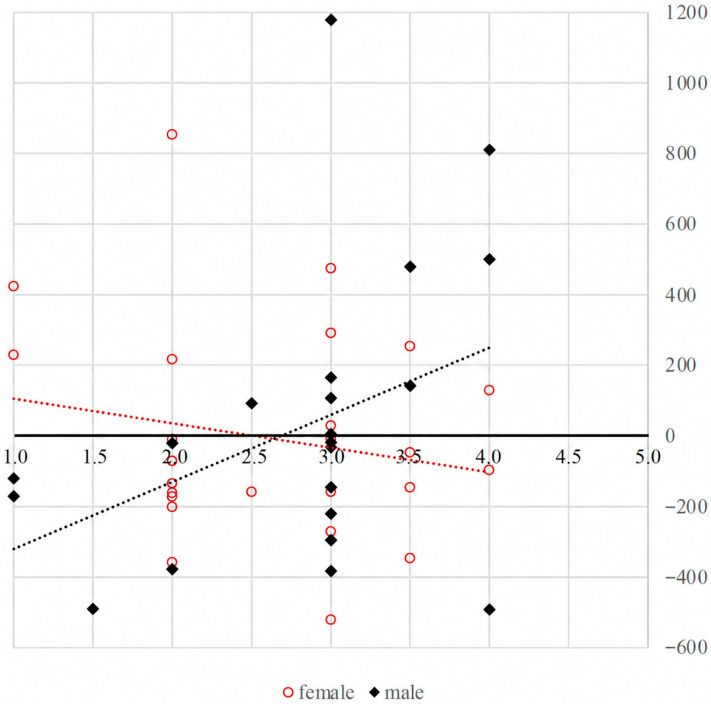
Scatterplot showing the association between self-rated confidence in visual blood-loss estimation (5-point Likert scale) and cumulative absolute estimation error (mL). Each point represents one participant. Confidence was averaged across all four simulation scenarios. Dash lines represent linear regression trends for each gender.

Figure 2 illustrates the relationship between self-rated confidence and absolute estimation error separately for women and men. Each data point represents an individual participant, with their cumulative absolute estimation error in mL of all four scenarios on the *y*-axis and confidence ratings averaged across all four scenarios on the 5-point Likert scale on the *x*-axis. These parameters reflect overall perceived certainty and overall estimation accuracy.

Among female participants, confidence was not associated with estimation accuracy (Spearman ρ = −0.016, *p* = 0.934). In contrast, among male participants, higher confidence was significantly associated with larger estimation errors (Spearman ρ = 0.430, *p* = 0.046).

This gender-specific discrepancy reflects a calibration gap: women’s confidence levels appeared well aligned with their actual performance, while men tended to be overconfident, particularly at higher levels of self-rated certainty.

### 3.4. Subgroup Analyses and Error-Direction Patterns

Exploratory subgroup analyses found no significant association between estimation accuracy and menstrual cycle status, age, or years of clinical experience. Women who reported having current menstruation cycles did not differ significantly from those with no cycle in terms of absolute error. Similarly, greater years of clinical experience were not associated with improved estimation accuracy overall. There was, however, a non-significant tendency for more experienced anesthetists to show somewhat larger overestimations, although this trend did not reach statistical significance.

Error-direction analysis examined whether clinicians systematically underestimated or overestimated blood loss across scenarios. The observed pattern was consistent with previously described volume-dependent biases in visual blood-loss estimation. In low-volume scenarios, underestimation predominated across all participants, reflecting the well-known tendency to visually underrate smaller quantities of blood. In contrast, overestimation became more prominent in high-volume scenarios, particularly in Scenario 4. This effect was most pronounced among anesthetists, who frequently produced large positive deviations from the true blood-loss volume.

## 4. Discussion

This study examined how gender and professional background influence the accuracy of visual blood-loss estimation across four standardized obstetric hemorrhage simulations. The findings demonstrate that gender-related differences emerge primarily in low and moderate-volume scenarios, whereas professional background exerts a consistent and robust influence across all bleeding conditions. Women estimated blood loss more accurately than men in two of the four scenarios, particularly when tasks required the discrimination of subtle visual cues. Gynecologists consistently outperformed anesthetists and displayed substantially lower variability in estimation errors.

The observation that women performed more accurately in low-volume conditions aligns with evidence from the science demonstrating gender differences in fine-grained visual perception and processing. Prior research shows that women outperform men in contrast sensitivity, color discrimination, and the detection of subtle luminance gradients—skills that may be especially important in early or less pronounced bleeding where visual signals are less salient [5,7]. Scenarios 1 and 3 exemplify this pattern, with women demonstrating both lower errors and tighter clustering of estimates.

In high-volume settings, gender differences diminished. In Scenario 4, women and men showed nearly overlapping estimation performance. One plausible explanation is visual saturation: as blood volume increases, the perceptual challenge shifts from interpreting fine visual details to judging large fluid collections. Under such conditions, clinicians, irrespective of gender, may rely on global heuristics rather than subtle visual cues. The existing literature notes that both men and women tend to apply size-based heuristics when interpreting large volumes, often resulting in overestimation [3].

Professional background emerged as a stronger predictor of estimation accuracy than gender. Gynecologists produced consistently lower errors across nearly all scenarios. Their narrower error distributions suggest a more uniform perceptual calibration, likely developed through repeated intraoperative exposure to obstetric bleeding. This finding is consistent with prior simulation and clinical studies indicating that obstetricians and surgeons outperform clinicians whose practice relies less on direct visual blood assessment. In contrast, anesthetists generally depend on physiological indicators such as hemodynamic changes and laboratory values. The absence of such cues in this simulation environment may have contributed to their wider variability of estimates.

The error-direction patterns further support these interpretations. Women demonstrated balanced tendencies for underestimation and overestimation in low-volume settings, while men more often underestimated small volumes. Anesthetists, meanwhile, showed substantial overestimation in high-volume scenarios. These volume-dependent biases are well-documented in the literature and likely reflect a combination of visual dilution and expectation-driven heuristics [2,8,9].

Exploratory subgroup analyses found no association between estimation accuracy and menstrual status, age, or years of experience. Although hormonal fluctuations have been shown to influence attentional or visuospatial performance in some controlled experiments, our findings indicate no clinically meaningful effect in visual blood-loss estimation. Similarly, years of professional experience did not correlate with improved accuracy overall. The tendency toward greater overestimation among more experienced anesthetists may reflect increased vigilance or risk-averse practice, rather than improved perceptual accuracy.

The relationship between confidence and performance offers additional insight into gender-related patterns in clinical perception. At the overall cohort level, confidence was not associated with accuracy. However, gender-stratified results revealed marked differences: women displayed well-calibrated metacognition, with confidence levels closely reflecting actual performance. In contrast, men showed a positive correlation between confidence and error, indicating overconfidence. This phenomenon is consistent with the previous literature, showing that male clinicians often exhibit higher self-assessed certainty, which does not reliably predict performance in diagnostic and procedural tasks [1,7,10].

Our findings underscore that there is risk of overconfidence with visual blood-loss estimation that may delay escalation during hemorrhage when clinicians overestimate the accuracy of their assessments. Individualized feedback during simulation may help mitigate these behaviors [11].

Targeted simulation-based training seems especially important for clinicians with limited visual exposure to obstetric bleeding and may help reduce estimation errors and improve situational awareness. Incorporating feedback mechanisms that strengthen perceptual calibration and address overconfidence may further enhance the accuracy of hemorrhage assessment [12].

### Strengths and Limitations

This study has several strengths, including balanced gender and professional representation, the controlled presentation of realistic hemorrhage scenarios, verified hemoglobin concentrations, and standardized viewing conditions. The methodology allows for the direct examination of gender or profession-specific perceptual influences. Nonetheless, limitations must be acknowledged. Simulations cannot fully replicate the complexity of real obstetric hemorrhage, where visual cues interact with dynamic bleeding, tissue appearance, and changing vital signs. The absence of physiological information in this simulation may disadvantage certain professional groups, particularly anesthetists. Furthermore, although the sample size is comparable to other simulation studies, it may have limited our ability to detect smaller gender effects. Finally, the findings may not generalize across institutions with different training practices or visual exposure to obstetric hemorrhage.

## 5. Conclusions

This simulation-based study indicated that both gender and professional background may influence the accuracy of visual blood-loss estimation. Women showed higher accuracy than men in low and moderate-volume scenarios. Professional background proved to be a statistically stronger determinant of accuracy: gynecologists consistently outperformed anesthetists across nearly all scenarios and exhibited markedly less variability, indicating more reliable perceptual calibration gained through repeated clinical exposure to obstetric bleeding. These findings underscore that visual blood-loss estimation is influenced by a combination of perceptual abilities and experiential familiarity. Due to the small sample size and the nature of the study involving simulated scenarios, our results show a tendency that needs to be confirmed by further and larger studies.

Nonetheless, these results highlight the importance of interdisciplinary communication during hemorrhage management. Improving clinical safety in obstetric hemorrhage requires an interdisciplinary approach that accounts for systematic performance differences between clinician groups. Targeted simulation-training strategies could help to address both gender-related and profession-related perceptual biases.

Overall, integrating these insights into obstetric training and team communication practices may contribute to the more effective and timely management of obstetric hemorrhage.

## Figures and Tables

**Table 1:** Participants’ characteristics, professional background, clinical experience, and self-rated confidence.

Participants’ Characteristics	Total (*n* = 50)
Male gender, *n* [%]	22 [44%]
Active menstrual cycle, *n* [%]	0 [0%]
Female gender, *n* [%]	28 [56%]
Active menstrual cycle, *n* [%]	23 [82%]
Anesthesiology, *n* [%]	25 [50%]
Gynecology, *n* [%]	25 [50%]
Clinical experience (years), median (IQR)	6.0 [7.5]
Self-rated confidence (1–5 scale), mean ± SD	2.6 ± 1.0

**Table 2:** Scenarios with clinical contexts and blood compositions.

Scenario	Clinical Context	Final Volume [mL]	Measured Final Hb [g/dL]	PRBC Volume [mL]	NaCl Volume [mL]	PRBC:NaCl Ratio
1	Caesarean birth	438	10.9	239	199	1:0.83
2	Ectopic pregnancy	811	9.4	381	430	1:1.13
3	Placenta previa	622	8.6	268	354	1:1.32
4	PPH	1014	6.5	330	684	1:2.07

Hb—hemoglobin, NaCl—sodium chloride solution, PRBC—packed red blood cells.

## Data Availability

Data is contained within the article or supplementary material.

## Supplementary Materials

The following supporting information can be downloaded at: https://www.mdpi.com/article/10.3390/jcm15135142/s1, Figure S1: Scenario Photos; Table S1: Absolute estimation error by gender and professional background.

## Abbreviations

The following abbreviations are used in this manuscript:

EBL	Estimated blood loss
PPH	Postpartum Hemorrhage
PRBC	Packed Red Blood Cells
