# Supplementary material for: The Influence of Gender and Professional Background on the Accuracy of Visual Blood-Loss Estimation in Obstetrics—Prospective Observational Simulation Study
Source: J Clin Med. 2026 Jul 1;15(13):5142. doi: 10.3390/jcm15135142 (PMC13363368; doi:10.3390/jcm15135142)
Supplement: Supplementary file 1 [file jcm-15-05142-s001.zip › jcm-4353504-Table S1.pdf]

Supplementary Table S1. Absolute estimation error by gender and professional background across obstetric haemorrhage simulation scenarios

Scenario (true blood loss)	Group	Median absolute error (mL)	IQR (mL)	Maximum absolute error (mL)
Scenario 1 (438 mL)	All participants	162	193	762
	Women	88	113	410
	Men	225	133	762
	Gynaecologists	62	124	438
	Anaesthetists	238	156	762
Scenario 2 (811 mL)	All participants	261	278	1189
	Women	211	179	901
	Men	311	368	1189
	Gynaecologists	211	200	901
	Anaesthetists	311	292	1189
Scenario 3 (622 mL)	All participants	222	250	878
	Women	150	211	622
	Men	300	311	878
	Gynaecologists	128	194	622
	Anaesthetists	372	354	878
Scenario 4 (1014 mL)	All participants	364	354	2486
	Women	375	320	1720
	Men	350	387	2486
	Gynaecologists	264	300	1410
	Anaesthetists	486	350	2486

Absolute estimation error represents the absolute difference between visually estimated and true blood-loss volume. Values are presented as medians with interquartile ranges (IQR) and maximum absolute deviations.
