# Supplementary figures and images for: The Influence of Gender and Professional Background on the Accuracy of Visual Blood-Loss Estimation in Obstetrics—Prospective Observational Simulation Study
Source: J Clin Med. 2026 Jul 1;15(13):5142. doi: 10.3390/jcm15135142 (PMC13363368; doi:10.3390/jcm15135142)

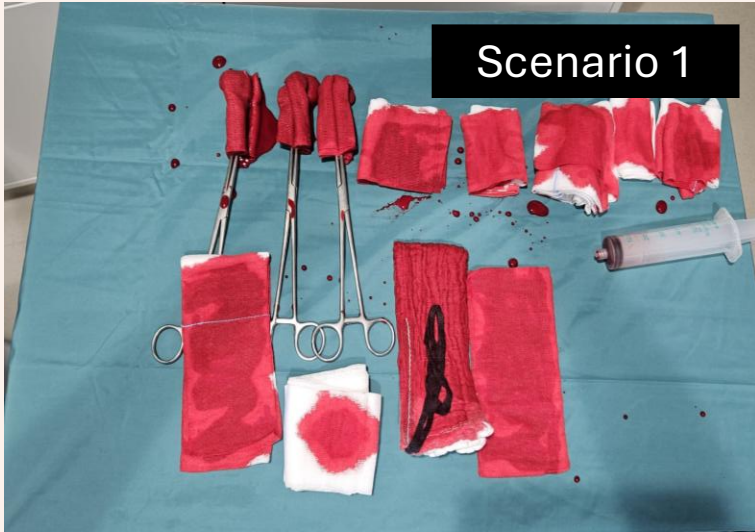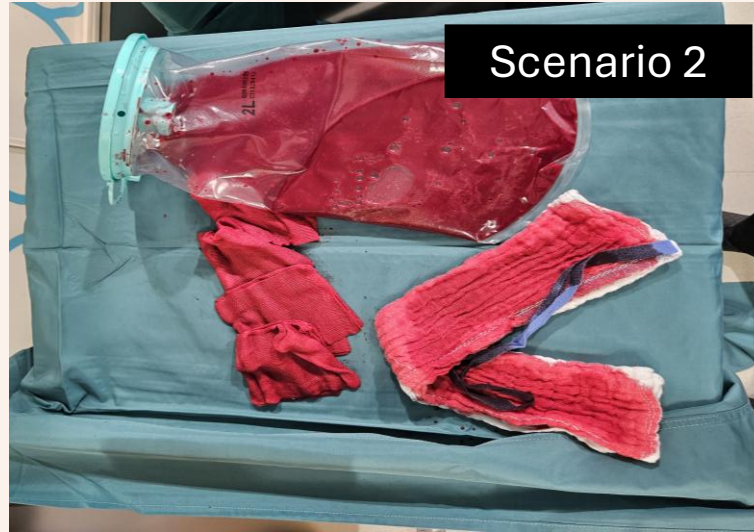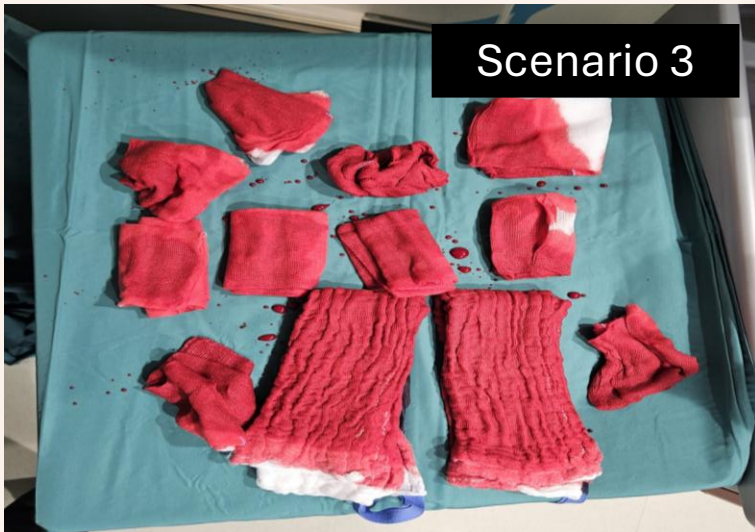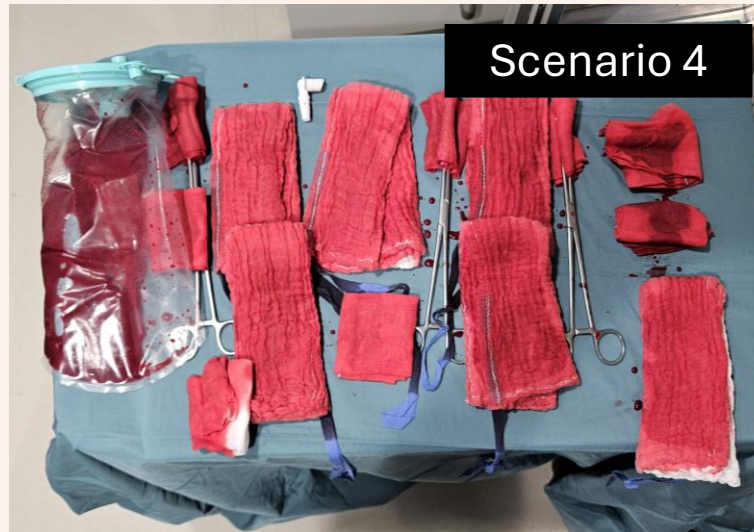

Supplement: Supplementary file 1 [file jcm-15-05142-s001.zip › jcm-4353504-Supplementary Scenario Figure S1.pdf]
